# IDENTIFYING UNMET NEED IN INFORMAL CAREGIVING: DISPARITIES BY GENDER, EMPLOYMENT STATUS, AND RACE-ETHNICITY

**DOI:** 10.1093/geroni/igz038.803

**Published:** 2019-11-08

**Authors:** Steven A Cohen, Furong Xu, Marissa R Meucci, Symone Woodham, Mary L Greaney

**Affiliations:** University of Rhode Island, Kingston, Rhode Island, United States

## Abstract

Older adults, including those with dementia and other types of cognitive decline, often report a desire to remain in their homes. Over 50 million informal caregivers in the US provide needed in-home assistance to those in need, and there are well-documented disparities in informal caregiving responsibilities by sociodemographic factors , yet little is known about “unmet need” in informal caregiving. Therefore, the study’s objective is to examine discrepancies in unmet caregiving-related need by race/ethnicity, gender, and employment status. We abstracted data about caregivers from the 2017 National Study of Caregiving and linked these data to participants in the National Health and Aging Trends Study on caregivers of older adults (n=993). Generalized linear models were used to model the discrepancies between the number of activities of daily living for which the care recipient required assistance and the number of tasks caregivers provide, by race/ethnicity, gender, and employment status, accounting for confounders and complex sampling. Care recipients whose primary informal caregivers were employed were 69% more likely than those whose informal caregivers were not employed to experience unmet caregiving need (OR 1.69, 95%CI 1.19-2.41). A similar association between employment and unmet caregiving was observed among White caregivers (OR=1.79, 95% CI 1.16-2.69), while the association was not significant among Black caregivers (p=0.228). These findings suggest potentially addressable disparities in informal caregiving duties between Black and White caregivers, and can be used to inform and develop of policies and programs designed to improve caregiver health and reduce undue strain on caregiver health and wellbeing.

